# The Cancer and Work Scale (CAWSE): Assessing Return to Work Likelihood and Employment Sustainability After Cancer

**DOI:** 10.3390/curroncol32030166

**Published:** 2025-03-14

**Authors:** Christine Maheu, Mina Singh, Wing Lam Tock, Jennifer Robert, Andrea Vodermaier, Maureen Parkinson, Naomi Dolgoy

**Affiliations:** 1 Ingram School of Nursing, McGill University, Montréal, QC H3A 2M7, Canada; jennifer.robert2@mail.mcgill.ca; 2 School of Nursing, York University, Toronto, ON M3J 1P3, Canada; minsingh@yorku.ca; 3 Centre de Recherche, Centre Hospitalier de L’Université de Montréal, Montréal, QC H2X 0A9, Canada; wing.lam.tock@umontreal.ca; 4 Department of Social and Preventive Medicine, École de Santé Publique, Université de Montréal, Montréal, QC H3N 1X9, Canada; 5 School of Population and Public Health, University of British Columbia, Vancouver, BC V6T 1Z4, Canada; andrea.vodermaier@ubc.ca; 6 BC Cancer, Vancouver, BC V5Z 4E6, Canada; mparkins@bccancer.bc.ca; 7 Department of Occupational Therapy, University of Alberta, Edmonton, AB T6G 2G4, Canada; dolgoy@ualberta.ca

**Keywords:** return to work, cancer, work reintegration, tool validation, oncology vocational rehabilitation, exploratory factor analysis, work sustainability, cancer survivorship, work-related barriers, employment outcomes

## Abstract

**Background:** Returning to and sustaining employment after cancer presents significant challenges for individuals touched by cancer (ITBC). While vocational rehabilitation and workplace accommodations are critical, existing return to work (RTW) assessments lack cancer-specific considerations, limiting their clinical and occupational utility. **Purpose:** This study aimed to develop and validate the Cancer and Work Scale (CAWSE), a psychometrically robust tool designed to assess RTW likelihood and employment sustainability among ITBC, while also providing avenues for targeted interventions. **Methods:** A two-phase cross-sectional study was conducted. Study I (n = 130) assessed content validity and construct development, leading to a refined 43-item CAWSE. Study II (n = 216) employed exploratory and confirmatory factor analyses to establish structural validity, reliability, and responsiveness. Additional validation included correlations with fatigue, cognitive difficulties, depression, and anxiety. **Results:** Factor analysis supported a seven-factor structure with 31 final items. The CAWSE demonstrated good internal consistency (α = 0.787), construct validity, and moderate responsiveness (AUC = 0.659). High sensitivity allowed for accurate identification of RTW difficulties, with an established cut-off score of 123.5 on the total CAWSE. **Implications:** The CAWSE fills a critical gap in oncology-specific vocational rehabilitation, offering healthcare providers a validated tool for targeted interventions to enhance RTW outcomes and long-term employment sustainability for ITBC.

## 1. Introduction

### 1.1. Background

Sustaining employment and returning to work after cancer are crucial elements of quality care in cancer survivorship. Many individuals touched by cancer (ITBC) perceive vocation as representative of health recovery and a return to normalcy [1,2]. Furthermore, employment offers a sense of productivity and financial independence, mitigating the social isolation and negative identity shift often experienced as a result of not working during the cancer and treatment period [3].

There are strong indicators of the challenges associated with working following cancer diagnosis and treatment. A cancer diagnosis carries an elevated risk of adverse work-related outcomes, including unemployment, job loss, reduced working hours, early retirement, and being less likely to be re-employed [4,5,6,7]. Research shows that ITBC frequently report experiencing varying degrees of cancer-related and treatment side effects, some of which can be debilitating. These cancer-related side effects can encompass extreme fatigue, physical and cognitive exhaustion, emotional distress, anxiety, cancer-related pain, and a sense of physical, emotional, and cognitive deconditioning to meet the work demands [2,4,5,6,7]. To date, there is no assessment tool specifically designed to address the intersection of cancer and work that encompasses aiding in the identification of an individual’s potential to maintain employment following cancer and providing assessment-driven guidance for targeted interventions. Prior initiatives aimed at developing Patient-Reported Outcome Measures (PROMs) for ITBC have been limited, often focusing on cancer symptoms and physical outcomes [8], overlooking the intricate dynamics and complexities associated with cancer and work, particularly in the context of sustained employment [9,10,11]. Several general return to work (RTW) tools are available, but their applicability to cancer populations is limited. For example, two widely used assessments for general musculoskeletal issues include the Readiness to Return to Work (RRTW) tool [12] and the Return-to-work Obstacles and Self-Efficacy Scale (ROSES) [13], do not consider or assess for factors related to cancer, cancer sequelae, treatment-related considerations, and post-cancer return employment retention [14].

While some work-readiness and work-related screening tools specific to cancer-related work issues exist, these tools have limited approaches. Specifically, the Successful Return-to-work Questionnaire for Cancer Survivors (I-RTW_CS) [11] and the Quality of Working Life Questionnaire for Cancer Survivors (QWLQ-CS) [15] are tailored for cancer populations. But these are limited in their capabilities to address both individual and employer issues, and in offering any guidance for tailoring interventions. The I-RTW_CS aids employers but overlooks factors experienced by ITBC, such as workplace dynamics and identity shift and values; while the QWLQ-CS assesses for interventions but excludes issues like workplace discrimination and stigma [15]. Overall, tools for assessing work engagement for ITBC have yet to explicitly integrate oncological vocational rehabilitation factors, which are well-recognized for their influence on an individual’s capacity to sustain employment post-cancer into the assessment or guidance [11,16,17,18,19]. Often omitted factors encompass the meaning of work, financial stress, social pressures, and the work contexts [1,20,21]. Thus, there is a pressing need for the development and refinement of RTW tools specifically designed for ITBC. These tools should not only incorporate known vocational and oncological factors that influence RTW and sustained employment but also expand upon existing measures to provide a more comprehensive assessment of ITBC’s likelihood to successfully return to and maintain employment.

### 1.2. Factors Associated with Sustained Employment and RTW in ITBC

**Figure 1:**
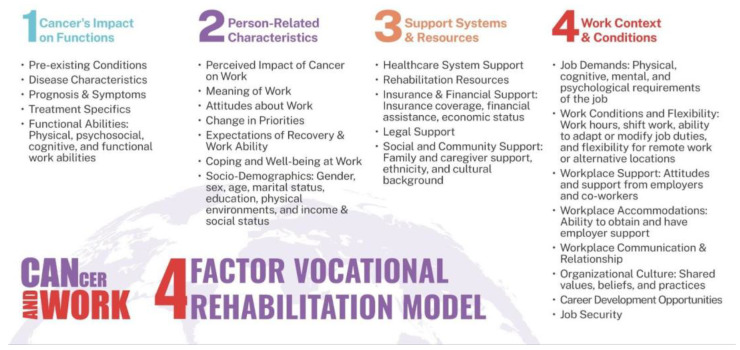
Cancer and Work: Four Factors Vocational Rehabilitation Model for Individuals Living with Cancer. Footnote: Revised from the original model, “Vocational Rehabilitation Model for Cancer Survivors” [3].

Following a cancer diagnosis, several factors have been found to influence the likelihood to sustain employment, including RTW. A recent meta-synthesis analysis identified three overarching themes: person-related, employment-related, and wider contextual factors, which included family, social, and cultural influences [1]. Aligning with these themes, the Four Factor Vocational Rehabilitation Model was developed through an evidence-informed, stakeholder-engaged process specific to cancer survivorship [3,22,23]. In addition to reflecting these themes, the model introduces a fourth domain—cancer’s impact on functions—to comprehensively address the unique challenges faced by ITBC [16,24]. Figure 1 outlines the Four Factor Vocational Rehabilitation Model:

Factor 1—Cancer’s Impact on Functions–evaluates the ramifications of cancer and its associated treatments on an individual’s physical, psychological, and cognitive functions.

Factor 2—Person-Related Characteristics–examines individual attitudes toward work and recovery within specific socio-demographic contexts (such as age, gender, and earnings).

Factor 3—Support Systems and Resources–assesses the availability and efficacy of support systems for employment sustainability, including healthcare and insurance provisions.

Factor 4—Work Context and Conditions–appraises job demands in relation to an individual’s work ability post-cancer, considering workplace adaptations and organizational culture.

While independent of the World Health Organization International Classification of Functioning, Disability and Health (WHO’s ICF) framework, the model shares conceptual alignment in its emphasis on person-related and environmental factors that influence RTW for ITBC [25].

### 1.3. Study Purpose

Understanding the complex interplay of cancer on work, and the limitations of current tools available, the purpose of Study I and II is to bridge the gap in validated RTW measures in ITBC by developing and validating the Cancer and Work Scale (CAWSE). The CAWSE is designed to assess individuals’ likelihood to RTW and sustain employment after cancer, covering a broad spectrum of work scenarios including returning to work, current working, short-term work conditions and sustaining employment. The assessment is sensitive to modifications and adaptations associated with RTW after cancer. An additional aim of this study is to equip healthcare providers with the ability to offer tailored support aligned to the unique challenges that ITBC face in RTW and work maintenance. Item selection in the CAWSE was designed to provide intervention guidance and strategies for enhancing and sustaining employment specific to cancer-related challenges.

### 1.4. Ethics Statement

The study was approved by the Research Ethics Board at the University Health Network (REB #12-5184-CE), which was the primary research institute affiliation of the first author, CM, at the time of conducting this study. Written informed consent was collected from all of the subjects. Oral and written information about the study was given to all potential participants. The information provided to prospective participants included the purpose of the study, the possibility to withdraw at any point without any effects on current or future treatment, and the assurance that data would be presented only on a group level and that all individual data would be anonymized.

## 2. Materials and Methods

The development and evaluation of the patient-reported outcome measure (PROM), CAWSE, adhered to the standards and guidelines outlined in the COSMIN (COnsensus-based Standards for the selection of health Measurement INstruments) manual. This manual provides a comprehensive framework for assessing the methodological quality of studies examining the measurement properties of health-related PROMs [26,27,28]. The development and evaluation process of the CAWSE occurred in two distinct studies. Study I aimed to assess the content validity of CAWSE focusing on PROM development (COSMIN Standard 1) and on the first of nine COSMIN measurement property assessments: content validity (Standard 2). Study II aimed to evaluate the internal structure of the PROM in alignment with COSMIN Standards 3 to 5, as well as the remaining measurement properties outlined in Standards 6 to 10 [29]. The specific contributions of each study to the development and evaluation of CAWSE are detailed below.

### 2.1. Study I—Participants of the Content Validation Procedures of Item Development and Content Validation

The iterative process of item development and content validation is summarized in Table 1. The initial development of the PROM’s items required participation from professional experts and from patient partners. The professional expert group included a member of our author group (AV) and four content experts, including cancer patient-partners and health professionals with oncology research and clinical practice backgrounds with at least 10 years of clinical and/or research experience. Patient partners refers to ITBC recruited through cancer centre databases and/or university research teams who participated in the development process. The CAWSE items were developed using a formative model, which aligns with previous scale developments focusing on disease and work [11,30]. This model builds constructs from individual items without assuming that changes in one area necessarily correlate with changes in another. For example, an improvement in fatigue symptoms is not presumed to result in higher satisfaction at work. The professional expert group provided feedback and recommendations to support the initial item generation and scale development, maintaining constructive involvement throughout the process.

The development process began with a comprehensive review of the literature addressing factors aiding and hindering sustained employment and RTW after cancer in both employed and self-employed individuals. The literature review was complemented by ongoing focus groups and feedback sessions with the professional expert group. Through this iterative process, recurring themes across current assessments and publications were identified. Guiding questions for the literature review and focus groups in Study I included inquiries about the scope and evolution of symptoms reported after resuming work, the types of workplace accommodations utilized, the role of returning to work as a coping strategy, the significance of work and how this perception may change over time, and the influence of financial factors after returning to work.

Responses to these guiding questions, gathered from the literature and feedback sessions, informed the initial items generation. Recurring items were flagged, aggregated, and redundancies were eliminated based on consensus among the professional experts and patient partners. The final review yielded 16 items, which then underwent content validation via an online survey administered to ITBC. The purpose of the content validation was to ensure that the items included in CAWSE were conceptually relevant, comprehensive, and clear for ITBC [31], thereby enhancing the measure’s overall validity and applicability in assessing likelihood to RTW and sustain work after cancer.

### 2.2. Study I—Participants of the Content Validation

Item generation development of the PROM (Standard 1) included participation from the professional expert group. For content validation (Standard 2) of the retained 16 items, ITBC who had completed primary cancer treatment within the past year were identified from a cancer centre database in British Columbia, Canada. Eligible participants were mailed an invitation to participate and provided with a study questionnaire package, which included the 16-item survey designed to assess the tool’s validity. The recruitment process was further supported at the cancer centre through on-site posters, general emails, and word-of-mouth referrals. Inclusion criteria specified individuals aged 18 to 65 years who were English-speaking, had been employed prior to their cancer diagnosis, and intended to return to work following treatment.

#### 2.2.1. Study I—Variables and Measures

The initial item generation for the PROM-CAWSE resulted in a 16-item survey, with responses captured using a 7-point Likert scale ranging from *Strongly Disagree* to *Strongly Agree*. Examples of these items include: “*What I do outside my work became more important to me*”, “*I need to work because I depend on the income*”, and “*If I do not work, I will be lonely*”.

To assess the relevance and validity of these items, the online survey included the CAWSE alongside four other validated measures: (1) the Anxiety and Depression subscales of the PSSCAN [32,33], each containing five items; (2) the 4-item Fatigue subscale of the SF-36 Vitality Subscale (SF-36-F) [34]; and (3) five items from the Assessment of Survivor Concerns (ASC) [35], which measures fear of cancer recurrence and concerns about ill health.

This process, which involved testing the generated CAWSE items against validated instruments to assess their relevance and construct validity, aligns with the COSMIN methodology for evaluating content validity [31].

#### 2.2.2. Study I—Analysis Plan

To assess the content validity, concurrent and divergent validity were examined by calculating correlation coefficients between the CAWSE and the PSSCAN anxiety and depression subscales, the SF-36 fatigue subscale, and the five items ASC. The Shapiro–Wilkinson test was conducted to assess the normality of the data distribution. If the data were normally distributed, Pearson’s correlation coefficient was used; otherwise, Spearman’s correlation coefficient was applied.

The evaluation of the measurement methodology was conducted through an assessment of structural validity, employing Principal Component Factor Analysis (PCA) to explore the underlying factor structure and internal consistency reliability, measured using Cronbach’s alpha (α). This methodological approach ensured that the CAWSE’s internal structure was appropriately examined in alignment with psychometric best practices.

Following an initial review of the content validity data (with results presented in the Results section of this manuscript), the expert focus group reconvened to determine whether the scale sufficiently captured the intended construct. Through this iterative refinement process, it was concluded that additional items were needed to enhance construct coverage. As a result, 27 items were added to the initial 16, resulting in a 43-item version of the CAWSE, the validation of which was undertaken in Study II.

### 2.3. Study II: Validation, Expansion and Application of the CAWSE Design and Development

Study II utilized an online survey methodology to validate and expand the CAWSE. The primary objective was to assess the reliability, construct validity, and applicability of the refined 43-item version of the scale. Additionally, the study aimed to examine the generalizability of the CAWSE by evaluating its psychometric properties in a distinct population from Study I. To enhance external validity, participants were recruited from a different cancer centre and a separate Canadian province, ensuring that the scale’s performance remained consistent across diverse clinical and demographic contexts.

#### 2.3.1. Study II—Participants

Participants were recruited through convenience sampling identified through a cancer centre database. Inclusion criteria required the participants to be: (1) 65 years or younger, (2) diagnosed with cancer within 2 years of taking part in the study, and (3) working prior to their cancer diagnosis. Exclusion criteria consisted of primary metastatic disease. Eligible participants received a letter informing them about the study. Participants who did not contact the study team were approached by phone and directed to a website or offered a direct link to the online questionnaire. Additionally, the questionnaire was designed to adapt item phrasing based on RTW status. Participants who had not yet returned to work received a version with future-oriented phrasing (e.g., “Working would make me feel like life goes on.”), whereas those who had already returned to work received a version formatted in the present tense (e.g., “Working makes me feel like life goes on.”).

#### 2.3.2. Study II—Variables and Measures

Aligned with COSMIN quality development of PROM, three domains were assessed– reliability, validity, and responsiveness–with each having specific measurement properties [23,26,28,33]. To evaluate the measurement properties of the CAWSE, use of validated scales included measures for depression, anxiety, fatigue, cognition, and cancer worry.

Depression was measured using the 7-item subscale of the Hospital Anxiety and Depression Scale (HADS) specific to Anxiety (A) and Depression (D) [36]. The HADS-A and HADS-D are screening tools widely used for detection of anxiety and depression. While this scale is not specific to cancer-related anxiety and depression, it has been extensively validated for use in cancer populations, and has been used to detect clinical levels of anxiety and depression and to validate new scales [37].

Fatigue and cognitive function were the primary domains assessed using the European Organisation for Research and Treatment of Cancer Quality of Life Questionnaire (EORTC QLQ-C30) [38]. The fatigue subscale of the EORTC QLQ-C30 includes three items evaluating fatigue experienced over the past week: “Did you need to rest?”, “Have you felt weak?”, and “Were you tired?”. Each item is scored on a four-point scale: “Not at all”, “A little”, “Quite a bit”, and “Very much”. In accordance with EORTC guidelines [39], responses were transformed to a 0–100 scale, with higher scores indicating more severe fatigue. Notably, a score of 40 or higher is considered clinically significant based on NCCN recommendations [40], consistent with other research findings [41]. Cognitive function challenges were evaluated using two specific items from the EORTC QLQ-C30 [38]: “Have you had difficulty remembering things?”, and “Have you had difficulty concentrating on things”.

Fear about cancer recurrence and ill-health was measured using the Assessment of Survivor Concerns (ASC) [35]. This well validated scale consists of 5 items, three of which capture cancer-related concerns and two health-related concerns. Usually, this scale contains 6 items, but the last item about the children of patients was dropped.

### 2.4. Study II—Data Analysis

The primary goal of Study II was to validate the 43-item CAWSE. The internal structure of the CAWSE was assessed using exploratory factor analysis (EFA) to examine the factor structure, followed by confirmatory factor analysis (CFA) to test the fit of the refined factor solution. To assess the model goodness of fit, several fit indices were utilized, including chi-square (χ^2^), root mean square error of approximation (RMSEA), and Akaike’s Information Criterion (AIC). All statistical analyses were conducted using SPSS version 26 (IBM Corporation, Armonk, NY, USA, 2022). Reliability was assessed through Cronbach’s alpha to determine internal consistency. Construct validity was evaluated using convergent and divergent validity (assessed through correlations with measures of fatigue [38], cognition [38], anxiety [36], depression [36], and fear of cancer recurrence and ill-health [35]. Known-group validity was obtained by comparing scores of cancer type and treatment modalities. Criterion validity was considered, but due to the lack of a gold standard for RTW assessment, it was assessed indirectly through comparisons with validated instruments. Responsiveness was examined through Receiver Operating Characteristic (ROC) curve analysis, with sensitivity and specificity evaluated for various cutoff scores to determine the optimal threshold for identifying individuals at risk of difficulty with work engagement and sustainability.

The evaluation of additional measurement properties included reliability, measurement error using the standard error of measurement (SEM) and Cohen’s D. Hypotheses testing for construct validity was conducted, including five key hypotheses. Hypothesis one tested convergent and divergent validity, positing that when work engagement increases, CAWSE scores and subscales will correlate with the Fatigue subscale. Hypothesis two examined convergent and divergent validity, suggesting that when work engagement increases, CAWSE scores and subscales will correlate with the Cognition subscale. Hypotheses three through five focused on divergent and discriminant validity. Hypothesis 3 proposed that when work engagement increases, CAWSE and its subscales will correlate with HADS-D. Hypothesis 4 stated that CAWSE and its subscales will not correlate with HADS-A. Hypothesis 5 posited that CAWSE and its subscales will not correlate with the ASC scale. The responsiveness of the scale was assessed using the area under the receiver operating characteristic curve (ROC) [42], with clinical performance of the CAWSE to identify RTW cases evaluated through sensitivity (true-positive rate) over 1-specificity (false-positive rate) over various cutoff scores to determine the optimal threshold for classification of identifying individuals at risk for lower likelihood in RTW and sustain employment [43,44].

## 3. Results

### 3.1. Study I—Demographics

In Study I, 438 ITBC completed the preliminary use of the CAWSE. Data from 130 participants were included in the final analysis, as these individuals were below 65 years of age and had been engaged in employment prior to their cancer diagnosis. The remaining 308 responses were excluded from the analysis, as these participants did not meet the criteria of being employed and below 65 years of age.

Among the 130 participants included in the analysis, 65.4% were female and 34.6% were male. The majority (71.5%) were married or in common-law relationships, while 27.7% were living alone. Most participants (82.3%) had a high school education or below, with 17.7% having pursued postsecondary education. Regarding ethnicity, 81.5% identified as Caucasian, and 17.7% identified as other ethnicities. Additionally, 74.4% of participants had children, while 25.6% did not. In terms of biomedical characteristics, breast cancer was the most common diagnosis (34.0%), followed by genito-urinary (19.1%), gynecological (13.5%), gastrointestinal (7.1%), and head and neck cancers (7.1%).

### 3.2. Study I—Content Validity: Scale Development

In Study I, the CAWSE was initially developed with 16 items to assess RTW issues after cancer. Factor analysis of these items revealed five subscales—Symptoms (six items), Workplace Accommodation (four items), Meaning of Work (two items), Coping (two items), and Importance of Work (two items)—collectively explaining 68.55% of the variance. Reliability varied across the subscales, with Symptoms demonstrating the highest internal consistency (α = 0.85, 22.69% variance), followed by Accommodation (α = 0.77, 15.78%), Meaning (α = 0.71, 11.51%), and Coping (α = 0.66, 10.39%). The Importance subscale had poor internal consistency (α = 0.38, 8.18%) and was excluded from further analysis due to insufficient reliability.

Construct validity was evaluated by correlating the remaining subscales with validated measures of anxiety, depression, fatigue, cancer worry, and health worry. The Symptoms subscale demonstrated significant correlations with anxiety (r = 0.34, *p* < 0.001), depression (r = 0.50, *p* < 0.001), cancer worry (r = 0.24, *p* < 0.05), and fatigue (r = −0.59, *p* < 0.001), reflecting its relevance to emotional and physical challenges in RTW. In contrast, Accommodation and Meaning showed weak, nonsignificant correlations with these measures, suggesting they assess distinct, non-health-related domains, such as workplace factors and personal values. The Coping subscale showed mixed results, correlating significantly with anxiety (r = 0.23, *p* < 0.05) but weakly with other measures.

Following this evaluation, an expert focus group identified gaps in the 16-item scale, including the underrepresentation of critical dimensions such as financial and health system factors, external workplace influences, and long-term coping mechanisms. To address these gaps, 27 additional items were developed, informed by participant feedback, findings from the literature, and insights from factor analysis, as well as relevance and validity data obtained through comparisons with the Anxiety and Depression subscales of the PSSCAN [32,33], the Fatigue subscale of the SF-36 Vitality Subscale [34], and the Assessment of Survivor Concerns (ASC) [35].

The resulting 43-item CAWSE, organized into four broader subscales, was carried forward for validation in Study II to ensure its robustness and applicability to diverse RTW contexts.

### 3.3. Study II—Demographics

The demographic and clinical characteristics of the 216 participants who completed the CAWSE in Study II highlight a predominantly Canadian-born sample (82%), with most participants identifying as White (79.9%), speaking English as their first language (83.6%), and holding a college degree (52.9%). Additionally, the majority were married or living with a partner (69.8%) and reported a household income greater than $80,000 (61.0%). Table 2 provides a detailed summary of these characteristics.

Clinically, 44.2% of participants had a diagnosis of breast cancer, while 26.5% were diagnosed with other cancers. The remaining cancer types each accounted for less than 10% of the sample. Most individuals had returned to work (76.1%) and were employed full time (80.8%). However, more than one-third of participants (38.0%) reported a change in their job position following their cancer diagnosis and treatment. These findings underscore the diverse backgrounds and RTW experiences of the participants, providing important context for interpreting the CAWSE results.

### 3.4. Study II—Internal Structure and Quality Assessment

The results for Study II are presented in accordance with the COSMIN framework, which recommends the following order: internal structure (including structural validity, internal consistency, and cross-cultural validity), followed by quality assessments of the scale (including hypothesis testing for construct validity, convergent and divergent validity, criterion validity and responsiveness). This structured presentation ensures that all key aspects of the CAWSE measurement properties are comprehensively addressed.

#### 3.4.1. Structural Validity

To determine the suitability of the data for factor analysis, the Kaiser-Meyer-Olkin (KMO) Measure of Sampling Adequacy was applied, yielding a value of 0.816, which indicated that the sample was adequate for factor analysis. Bartlett’s Test of Sphericity was significant (χ^2^(465) = 2634.501, *p* < 0.001), confirming the appropriateness of the correlation matrix for factor extraction [45,46].

The initial 43-item CAWSE underwent exploratory factor analysis (EFA) with Direct Oblimin rotation, which revealed an 11-factor structure explaining 69% of the variance. However, several items exhibited cross-loadings or had factor loadings below 0.30. To refine the factor structure, items with cross-loadings or low loadings were systematically reviewed by the authors, who are content experts in cancer and work-related issues. Redundant or conceptually inconsistent items were removed, leading to a refined seven-factor solution. This revised structure explained 66.45% of the variance, with all factors demonstrating acceptable internal consistency (Cronbach’s alpha: 0.619 to 0.899). The total scale showed good internal consistency, with a Cronbach’s alpha of 0.787. A scree plot confirmed the suitability of the seven-factor solution.

Confirmatory factor analysis (CFA) was conducted to evaluate the seven-factor structure. The CFA results supported the hypothesized model, with fit indices meeting acceptable thresholds: Comparative Fit Index (CFI) = 0.95, Tucker–Lewis Index (TLI) = 0.94, Root Mean Square Error of Approximation (RMSEA) = 0.06, and Standardized Root Mean Square Residual (SRMR) = 0.05. Factor loadings for individual items ranged from 0.41 to 0.91, indicating strong contributions to their respective subscales.

For Factor 7 (Meaning of Work), one item (“I would feel lonely if I was not working”) exhibited a factor loading slightly below the threshold (loading = 0.379). However, based on expert content review, it was retained due to its conceptual relevance. The internal consistency of the subscale remained acceptable (Cronbach’s alpha = 0.729), ensuring that the Meaning of Work domain was comprehensively measured.

**Figure 2:**
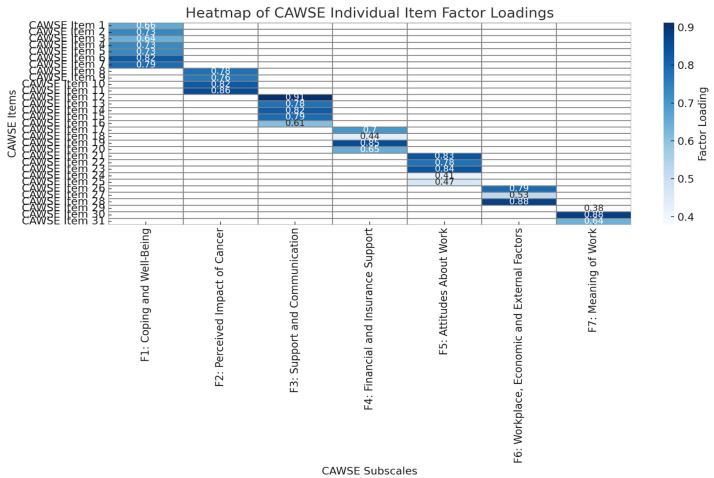
Heatmap of Individual Factor Loadings.

Factor loadings for all 31 items are visually represented in Figure 2. This visualization highlights item contributions to the seven subscales, with darker shades indicating higher loadings. The heatmap provides a clear overview of the CAWSE’s structural validity, reinforcing its robustness in measuring work engagement and return to work experiences for ITBC.

#### 3.4.2. Internal Consistency

Internal consistency of the CAWSE was evaluated using Cronbach’s alpha (α). The CAWSE demonstrated excellent internal consistency (α = 0.92). Subscale-specific reliability ranged from good to excellent, with Cronbach’s alpha values between 0.72 and 0.899 across most subscales, indicating that the items within these subscales reliably measure the intended constructs. For Factor 6 (Workplace, Economic, and External Factors), Cronbach’s alpha was 0.619, as shown in Table 3, which is slightly below the commonly accepted threshold of 0.7. However, this value was deemed acceptable given the exploratory nature of the scale and the diversity of the items included in this factor. Retaining all items ensured comprehensive coverage of the construct, which encompasses a broad range of workplace and external pressures. Moreover, expert review confirmed the conceptual relevance of the items in this factor, ensuring the integrity of the measurement. This decision highlights the importance of balancing statistical thresholds with clinical and conceptual considerations.

#### 3.4.3. Cross-Cultural Validity

Cross-cultural validity was assessed by comparing CAWSE scores across different cancer types and treatment modalities. Exploring whether individuals who received chemotherapy are less likely to return to work, a Chi-square test revealed no significant association (χ^2^(2) = 0.329, *p* = 0.848). Similarly, exploring whether individuals living with breast cancer are more likely to return to work compared to individuals living with other cancer types was also not supported (χ^2^(1) = 1.694, *p* = 0.193).

#### 3.4.4. Hypothesis Testing for Construct Validity

Construct validity was used to test the six hypotheses. For hypothesis one, the overall CAWSE score showed a significant negative correlation with fatigue (r = −0.332, *p* < 0.001), indicating that individuals with higher work engagement reported lower levels of fatigue. For hypothesis two, the results partially supported this hypothesis, as the overall CAWSE score was negatively correlated with cognitive function challenges (r = −0.261, *p* < 0.001), suggesting that higher work engagement was associated with fewer reported cognitive difficulties, although the correlation was weaker than that observed for fatigue. For hypothesis three, the results support this hypothesis, as demonstrated by a significant negative correlation between the overall CAWSE score and HADS-D scores (r = −0.314, *p* < 0.001); this finding suggests that individuals with fewer depressive symptoms tend to report better work engagement, greater coping, and more supportive workplace conditions. For hypothesis four, the overall CAWSE score showed no significant correlation with HADS-A (r = 0.089, ns), indicating that general anxiety levels do not substantially impact overall work engagement or support. For hypothesis five, the results did not strongly support this hypothesis, as the overall CAWSE score showed no significant correlation with ASC scores (r = −0.029, ns), suggesting that fear of cancer recurrence and ill-health appears to have limited direct influence on overall work engagement, as reflected by the non-significant correlation with the overall CAWSE score.

#### 3.4.5. Scale Quality: Convergent, Concurrent and Divergent Validity

**Figure 3:**
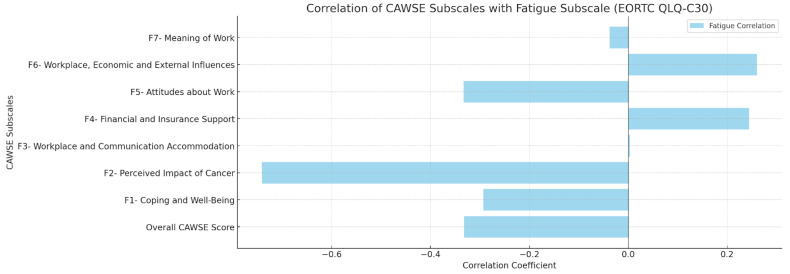
Concurrent Validity—Correlation of CAWSE Subscales with Fatigue Subscale (EORTC QLQ-C30).

**Figure 4:**
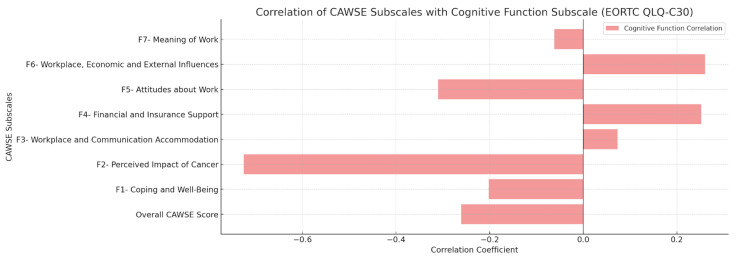
Concurrent Validity—Correlation of CAWSE Subscales with Cognitive Function Challenges (EORTC QLQ-C30).

Convergent validity was assessed by examining the correlations between CAWSE scores and the fatigue and cognitive function subscales of the EORTC QLQ-C30. The overall CAWSE score was negatively correlated with fatigue (r = −0.332, *p* < 0.001) and cognitive difficulties (r = −0.261, *p* < 0.001), supporting the hypothesis that higher work engagement is associated with lower fatigue and better cognitive function. Subscale-specific correlations for fatigue and cognitive function are visually presented in Figure 3 and Figure 4.

**Figure 5:**
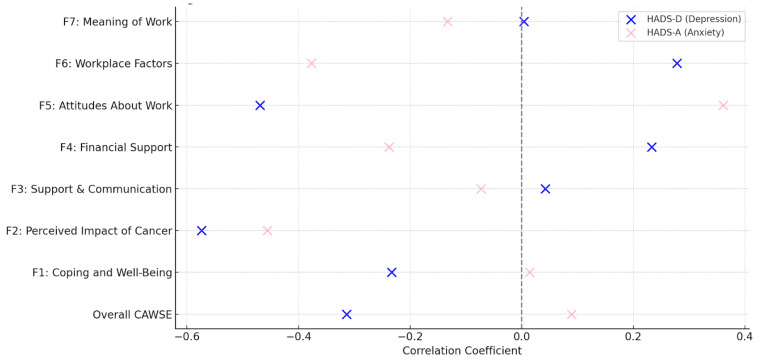
Scatter Plot of HADS-D and HADS-A vs. CAWSE Subscales.

Divergent validity was evaluated using the HADS-A and HADS-D subscales. The relationships between CAWSE scores and both HADS-D (depression) and HADS-A (anxiety) are presented in Figure 5.

Among the CAWSE subscales, F2: Perceived Impact of Cancer exhibited the strongest negative correlation with HADS-D (*r* = −0.574, *p* < 0.001), suggesting that individuals who perceive a greater impact of cancer on their ability to work also report higher depressive symptoms. Similarly, F5: Attitudes About Work (*r* = −0.469, *p* < 0.001) was significantly negatively correlated with HADS-D, indicating that positive attitudes toward work are associated with lower depressive symptoms. Interestingly, F4: Financial and Insurance Support showed a weaker, albeit significant, positive correlation with HADS-D (*r* = 0.233, *p* < 0.001), implying that financial pressures may contribute to depressive symptoms, despite individuals maintaining work engagement.

#### 3.4.6. Assessment of CAWSE Responsiveness

The responsiveness of the CAWSE was assessed using the area under the receiver operating characteristic (ROC) curve [42], focusing on the clinical performance of the scale in identifying RTW cases. The analysis evaluated sensitivity (true-positive rate) and 1-specificity (false-positive rate) across various cutoff scores to determine the optimal threshold for classifying individuals at risk of a lower likelihood of RTW or sustaining employment [43,44].

**Figure 6:**
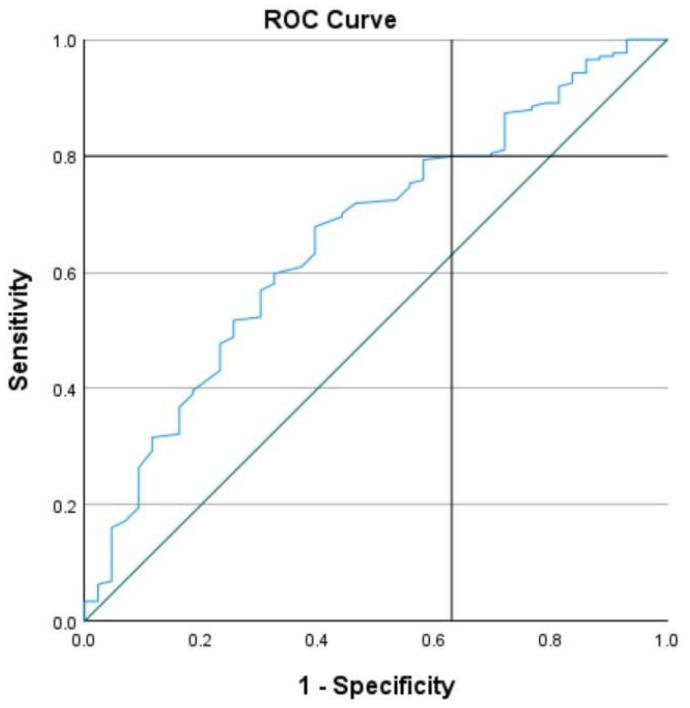
Receiver Operating Characteristic (ROC) Curve.

The ROC analysis revealed an area under the curve (AUC) of 0.659, indicating moderate discriminatory ability of the CAWSE to distinguish between individuals who RTW and those who did not. The optimal cutoff score was determined to be 123.5, which yielded a sensitivity of 79.9% and a specificity of 37.2% (see Figure 6). This threshold maximizes the scale’s ability to identify individuals at risk of experiencing difficulty or delay in RTW, prioritizing sensitivity to reduce the likelihood of false negatives. A total CAWSE score of 123.5 or below indicates a higher likelihood of requiring targeted interventions to address barriers to employment reintegration and sustain work. This is further supported by a “Yes” response to the final unscored question on the CAWSE, which flags additional concerns.

A detailed summary of sensitivity and 1-specificity values for scores surrounding the cutoff of 123.5 is provided in Table 4 below.

Cohen’s D effect sizes were also calculated to assess the magnitude of differences in CAWSE scores between those who returned to work (n = 174) and those who had not (n = 42). The total CAWSE score demonstrated a moderate effect size (Cohen’s D = 0.62), while subscale-specific effect sizes ranged from 0.03 to 0.96, with the highest differences observed in Meaning of Work (Cohen’s D = 0.96) and Attitudes About Work (Cohen’s D = 0.66). These findings indicate that the CAWSE is responsive in detecting meaningful differences in employment outcomes and identifying at-risk individuals for targeted support.

#### 3.4.7. Criterion Validity

No gold standard exists for measuring work engagement and return to work among ITBC; thus, criterion validity could not be directly established. However, the strong structural, convergent, divergent, and cross-cultural validity, along with good responsiveness, collectively support the CAWSE as a reliable and valid tool for assessing work engagement in ITBC.

## 4. Discussion

Returning to and maintaining employment after cancer presents multifaceted challenges [47]. Existing RTW tools fail to fully capture the complexity of oncological vocational rehabilitation, necessitating the development of the CAWSE. By incorporating psychosocial, economic, and workplace factors, the 31-item seven factor CAWSE tool provides a more comprehensive assessment of RTW readiness and employment sustainability for ITBC. A copy of the tool is provided in the Supplementary Materials (Supplemental Document S1) and may be reproduced with proper referencing.

### 4.1. Structural and Construct Validity

The CAWSE demonstrated a strong internal structure, with exploratory and confirmatory factor analyses supporting its seven-factor model. High internal consistency across subscales and acceptable fit indices confirmed the scale’s reliability. Construct validity was further established through significant correlations with fatigue, cognitive difficulties, and depression, reinforcing the CAWSE’s sensitivity to key employment-related barriers. However, workplace, economic, and external factors showed weaker associations with RTW outcomes, suggesting further refinement may be necessary to enhance predictive utility.

Convergent and concurrent validity tested with the EORTC QLQ-C30 [38], indicate that higher work engagement is associated with lower fatigue and better cognitive function. Divergent validity, assessed using the HADS-D and HADS-A [36], suggests that depression is more closely related to work engagement than anxiety, with higher depressive scores correlating with lower CAWSE scores. In contrast, anxiety appears to have a weaker and less consistent relationship with work engagement, although specific aspects of workplace-related anxiety—such as fear of job performance evaluation or social interactions—may still influence employment outcomes. While the relationship between work functioning and depressive symptoms in cancer patients is well established [48,49,50], the impact of workplace-related anxiety and psychosocial risks at work on RTW engagement remains less extensively studied [7].

The direct influence of Fear of Cancer Recurrence (FCR) and ill-health on overall work engagement appears to be limited, potentially due to contextual factors such as financial instability and lack of job security, which may overshadow the impact of FCR. Research suggests that the threat of cancer recurrence or progression poses significant challenges to financial security, making financial concerns a more critical factor in work-related decision-making than FCR alone [51]. However, more research is needed to accurately depict the interplay between these factors [47,52] and to determine whether enhancements to the CAWSE questionnaire, particularly in its items addressing workplace, economic, and external factors, might improve its ability to capture this dimension.

In this study, no significant association was observed between work return status and scores on the Factor 6 subscale, which assesses workplace, economic, and external factors. This finding aligns with the relatively low variance explained (4.91%) by this factor and its lower reliability (Cronbach’s alpha = 0.619); heterogeneity likely impacted the association. These results suggest that workplace, economic, and external factors may not strongly influence work engagement in this context, though further investigation is warranted to refine this subscale and enhance its relevance to employment outcomes among ITBC. This outcome is contrary to expectations given previous literature that highlights the negative association between a lack of workplace support and return to work after cancer. For instance, a systematic review by Mehnert et al. [6] found that workplace accommodations and support are crucial for successful RTW among ITBC. Such findings underscore the importance of further exploring workplace-related variables to better understand their role in employment outcomes for individuals affected by cancer. Moreover, this finding aligns with cross-cultural validity results, which suggest that factors beyond cancer type and treatment may play a more critical role in determining work engagement. This finding is notable given that previous evidence has consistently demonstrated that cancer type, stage, and treatments such as chemotherapy can significantly impact RTW outcomes. For example, research by de Boer et al. [7] found that individuals treated with chemotherapy, particularly for breast cancer or hematological cancers, often experience longer delays in returning to work and face greater challenges in sustaining employment. These insights underscore the complexity of employment outcomes for individuals touched by cancer, emphasizing the need for further investigation of workplace conditions and broader employment contexts.

### 4.2. Responsiveness

The CAWSE demonstrated good responsiveness in distinguishing between individuals who returned to work and those who did not, aligning with evidence that employability remains a significant challenge for ITBC even years after diagnosis. The high sensitivity ensures that most individuals at risk of not returning to work are accurately identified, which is critical for targeting appropriate interventions aimed at enhancing both occupational outcomes and health-related quality of life (HRQoL). While the moderate specificity may occasionally classify individuals who have returned to work as needing support, this trade-off is acceptable in clinical and occupational contexts [53]. Individuals scoring lower or near the CAWSE threshold of 123 may face ongoing challenges in sustaining employment, despite having resumed work. Research underscores that employment status is closely tied to HRQoL, and targeted interventions for individuals identified by the CAWSE can help address barriers to work reintegration and improve long-term outcomes, further emphasizing the tool’s utility in supporting sustained employability among ITBC.

### 4.3. The Need for CAWSE in Cancer-Specific RTW Assessments

Many existing RTW tools do not adequately capture the unique challenges faced by ITBC. Limitations in response formats and a lack of specificity regarding short-term, long-term, and late effects of cancer and its treatment are evident in currently available assessments. Many tools rely on binary (yes/no) responses, which fail to capture the nuanced and evolving nature of work-related difficulties among ITBC. For example, cognitive difficulties are a common yet transient post-treatment effect, with approximately 30% of ITBC experiencing cognitive impairments due to cancer and its treatment. However, these effects often improve within a year [54]. A binary response format may inaccurately categorize individuals as permanently impaired, whereas a tool incorporating graded responses can better reflect the fluctuating nature of work-related challenges and recovery. The CAWSE was developed to address these gaps by incorporating a Likert-scale response format, allowing individuals to indicate the extent to which they experience employment-related difficulties rather than selecting an all-or-nothing response. This ensures a more accurate and individualized assessment of RTW potential and employment sustainability over time.

### 4.4. CAWSE as a Dual-Purpose Tool for Assessing RTW and Employment Sustainability

For ITBC, RTW and sustaining work represents two interconnected yet distinct experiences. The CAWSE is uniquely designed to assess both situations, making it a flexible tool applicable to individuals at different stages of their employment journey. For individuals who have not yet returned to work, CAWSE evaluates concerns related to re-entering the workforce after cancer; perceived ability to return, including symptom management and work readiness; worries about work-related challenges, such as fatigue, cognitive difficulties, or the need for workplace accommodations to support reintegration; and the psychological coping mechanisms associated with the transition back to employment. For individuals who have already returned to work, CAWSE assesses the ability to sustain employment despite lingering symptoms or workplace challenges; concerns regarding work performance, productivity, and the impact of cancer-related changes; the effectiveness of workplace accommodations and adjustments in supporting long-term employment; and the ongoing meaning of work along with the role of coping strategies in maintaining employment.

To ensure relevance across different RTW statuses, the phrasing of CAWSE items is adjusted accordingly. Participants who have not yet returned to work receive items in a future-oriented format, while those who have already returned receive them in the present tense. For example, the item “Working would make me feel like life goes on.” (for those who have not yet returned to work) is reworded as “Working makes me feel like life goes on.” (for those who have already returned to work). This adaptive phrasing ensures that responses are applicable to each participant’s actual work status, enhancing the accuracy and interpretability of the CAWSE.

By capturing these distinct yet overlapping experiences, CAWSE functions as a dynamic tool for assessing both RTW potential and long-term work sustainability in ITBC. Future research should refine and expand CAWSE’s content to enhance alignment with specific vocational rehabilitation needs, further increasing its applicability in clinical and occupational settings. Further exploration of self-efficacy and confidence would be warranted to best utilize the CAWSE across populations of ITBC who are considering returning to work and for those who are already in process or actually working.

### 4.5. Comparison of CAWSE with Existing RTW Assessment Tools

Existing RTW tools in cancer populations do not comprehensively integrate key oncological vocational rehabilitation factors that influence sustained employment outcomes. The I-RTW_CS (Successful Return-To-Work Questionnaire for Cancer Survivors) [11], the Readiness for Return to Work (RRTW) Scale [12], the AVEM Occupational Stress and Coping Inventory [55], and the Work Limitations Questionnaire [10] primarily focus on work readiness, occupational stress, or work-related limitations. However, these tools fail to systematically integrate factors such as meaning of work, financial stress, or social pressures, which are elements critical to understanding long-term employability in cancer populations.

A more recent tool, the Adaptability to Return-to-Work Scale (ARTWS) [54], was developed to measure cancer patients’ adaptability in the RTW process. ARTWS assesses three key factors: focusing on rehabilitation, rebuilding self-efficacy, and adjusting plans [56]. While ARTWS provides valuable insights into work adaptability, it does not explicitly evaluate financial stress, workplace accommodations, job security, or career development opportunities. These missing components are essential in oncological vocational rehabilitation, further underscoring the need for a more comprehensive, cancer-specific RTW tool.

The Unique Contributions of CAWSE: AWSE is distinguished as a comprehensive and oncology-specific RTW assessment tool, uniquely incorporating factors such as meaning of work, financial concerns, and workplace accommodations—elements that are often missing in other RTW measures. While some refinements are needed, its dual-purpose design allows it to effectively assess both individuals who have returned to work and those who have not yet done so, making it a versatile and pragmatic tool in both research and clinical settings. Future expansions of CAWSE to align with the four-factor vocational rehabilitation model may further enhance its predictive ability and clinical relevance, ultimately improving its utility in supporting ITBC in their employment reintegration and sustainability.

### 4.6. Limitations

This research faced significant challenges due to the onset of the global COVID-19 pandemic in 2020, which delayed the completion of the study and introduced widespread changes in work practices. These shifts may necessitate updates and adaptations to the current version of the CAWSE to reflect the evolving nature of work environments and employment challenges faced by ITBC.

Regarding sample size adequacy, Study I exceeded the “Very Good” threshold outlined in the COSMIN guidelines [57], achieving 116% of the requirement with 130 participants, ensuring strong validity for the exploratory analyses conducted. However, the sample population had many female participants, which has potential for gender bias in our findings. Similarly, in Study II, 44% of participants had a breast cancer diagnosis, which likely resulted in a higher proportion of female respondents. This reflects a common trend in oncology research, where breast cancer populations are overrepresented. This gender imbalance may limit the generalizability of findings to male cancer survivors and those with non-breast cancer diagnoses. Additionally, our study did not collect data on whether participants received professional RTW support, such as vocational rehabilitation, counselling, or workplace accommodations. This may be a potential confounding factor influencing RRTW outcomes, as individuals with access to such services may have different trajectories than those without support. Future research should account for the role of professional interventions in shaping return to work success. For Study II, the sample size of 216 participants met the “Adequate” threshold but reached only 71.76% of the “Very Good” threshold. While not achieving the ideal standard for confirmatory factor analysis, the sample size is still within acceptable ranges for robust analysis. A larger sample size in future scale validation health, social, and behavioural studies would allow for more rigorous testing of the scale’s internal structure and greater generalizability of the findings [58].

Additionally, the CAWSE was developed based on an initial version of the current four factor vocational rehabilitation model for ITBC [23]. However, subsequent review of study II results by the current expert group of authors have highlighted that some elements within the four factor vocational rehabilitation model for ITBC are not well represented in the current version of CAWSE. Specifically, these include Factor 2: Perceived Illness Impact on Work, Abilities, and Attitudes—Missing components include illness perception, illness representations, and socio-demographic influences; Factor 3: Support System Resources—Limited representation of financial assistance and economic stability; and Factor 4: Work Context and Conditions—Limited coverage of job demands, work flexibility, organizational culture, career development opportunities, and job security. These findings highlight areas for potential enhancement of CAWSE in future iterations. By incorporating these missing elements, CAWSE could further increase its explanatory power and predictive validity for RTW and sustained employment outcomes. Further, the participants in this study were recruited from convenience sampling and reflect a more homogeneous group of working individuals, with high education and mostly White. Further research with larger, broader participant groups would ensure that the CAWSE is applicable across all demographics.

## 5. Conclusions

The CAWSE offers a comprehensive approach to understanding and addressing the factors influencing RTW and sustained employment among ITBC, and aids in the development of tailored interventions and support strategies to facilitate more successful return to or continued work stability after cancer. Future research and application of the CAWSE in context is essential in continuing to develop and refine the tool for ongoing use.

## Figures and Tables

**Table 1:** Development and Validation Process of the Cancer and Work Scale (CAWSE).

	Aim	Input	Decision	Output
**Study I: Content Validity**				
**1. PROM Development: Item Generation**				
Item building from a comprehensive review by five experts in return to work for individuals touched by cancer (ITBC). Identify recurring themes discussed during joint meetings. Independent item composition.	Build items that explore: Symptoms Workplace accommodations Return to work as a coping strategy Meaning of work Financial factors	Expert focus group assessment of the literature and formulation of initial items (n.b.: Expert group includes patient-partners).	Aggregation of items and elimination of duplicate items. Proofread items for clarity by uninvolved team members.	**Preliminary version of CAWSE** **16 items** on a 7-point Likert scale (range 0–6)
**2. Content Validity**				
**Content Validity Testing of the Preliminary Version of CAWSE with 130 participants**				
**Preliminary version of the 16-item CAWSE** with newly diagnosed cancer patients of less than 12 months. **Study I survey completed by 438 ITBC of which 276 (63%) were 65 or younger, and of these, 130 were working prior to cancer diagnosis.**	Test the provisional scale by evaluating patient responses, followed by a statistical analysis to systematically identify and select pertinent items for the final scale.	A second focus group with the initial five experts was held to review the factor loading of the 16-item CAWSE.	Following factor analysis, one item was removed due to low factor loading. Additionally, new items were created to improve alignment with the four-factor vocational rehabilitation model for individuals living with cancer. Hence, 28 new items were added to the 15 initial items.	Factor loading of the initial 16 items resulted in five subscales: Symptoms: 6 items Workplace accommodations: 4 items Meaning of work: 2 items Coping: 2 items Importance of work: 2 items **Cronbach alpha:** **Factor Analysis** Symptoms: 0.85 Accommodation: 0.77 Meaning of work: 0.71 Coping: 0.66 Importance of work: 0.38 Variance: 68.55% Final version of the PROM CAWSE with 43 items.
**Study II: Testing the final version of the questionnaire with a new population**				
**3 to 10. Evaluation of the Internal Structure and Other Measurement Properties According to COSMIN**				
Assessment of the 43 items CAWSE on its validity, reliability, and responsiveness. **Study II survey completion by 216 ITBC**	Test the psychometric properties of the CAWSE 43 items.	Based on the highest factor loadings observed in the data analysis, the 43 items were categorized into six factors.	***Internal Structure Validation*** **3.** **Structural Validity** - Factor Analysis **4.** **Internal Consistency** - Cronbach’s alpha **5.** **Cross-cultural and grouping validity** - Breast cancer vs. other cancer type - With and without surgery ***Remaining measurement properties*** **6.** **Reliability** **7.** **Measurement error** - Cohen D **8.** **Criterion validity** - Area under the curve (AUC) **9.** **Hypotheses testing for construct validity** **10.** **Responsiveness** Receiving operating characteristics (ROC)	

**Table 2:** Study II—Sociodemographic and Clinical Characteristics of Participants.

	n	%	Valid Percent
**Birth country (n = 189)**			
Canada	155	71.4	82.0
Other	34	15.7	18.0
**Ethnicity (n = 189)**			
White	151	69.6	79.9
Black	5	2.3	2.6
Asian	12	5.5	6.3
East Indian	5	2.3	2.6
Jewish	6	2.8	3.2
Other	10	4.6	5.3
**First language (n = 189)**			
English	158	72.8	83.6
French	6	2.8	3.2
Other	25	11.5	13.2
**Highest Education Level (n = 189)**			
High school	12	5.5	6.3
College	100	46.1	52.9
University	74	34.1	39.2
Other	3	1.4	1.6
**Relationship status (n = 189)**			
Single/Separated/Divorced/Widowed	57	26.3	30.2
Married/Living with a partner	132	60.8	69.8
**Total Household income (n = 187)**			
<40,000$	28	12.9	15.0
40,000$–80,000$	45	20.7	24.1
>80,000$	114	52.5	61.0
**Type of cancer (n = 181)**			
Breast	80	36.9	44.2
Hodgkin’s Lymphoma/Non-Hodgkin’s Lymphoma	16	7.4	8.8
Gynecological	11	5.1	6.1
Prostate and testicular	12	5.5	6.6
Thyroid	6	2.8	3.3
Kidney	5	2.3	2.8
Melanoma (3)/Other (48)	51	23.5	28.2
**Metastasis diagnosis (n = 176)**			
Yes	32	14.7	18.2
No	144	66.4	81.8
**Cancer treatment (n = 180)**			
Only received chemotherapy or radiation or adjuvant therapy	42	19.4	23.3
Combination of chemotherapy and/or radiation and/or adjuvant therapy with (84.4%) or without surgery (15.6%)	138	63.6	76.7
**Returned to work after diagnosis (n = 217)**			
Yes	174	80.2	80.2
No	43	19.8	19.8
**Change in position following return to work (n = 163)**			
Yes	62	28.6	38.0
No	101	46.5	62.0
**Type of work prior to cancer diagnosis (n = 214)**			
Full-time	173	79.7	80.8
Part-time	23	10.6	10.7
Homemaker (3)/Retired (1)	4	1.8	1.9
Working/Student full-time	12	5.5	5.6
Sick Leave or Disability	2	0.9	0.9

**Table 3:** CAWSE Factors with Cronbach’s Alpha and Cohen’s D.

Subscale	Explained Variance	Cronbach’s Alpha	Cohen’s D
Factor 1: Coping and Well-Being at Work	22.42%	0.899	0.34
Factor 2: Perceived Impact of Cancer on Work	15.15%	0.845	0.22
Factor 3: Support, Communication, and Accommodations at Work	8.53%	0.867	0.23
Factor 4: Financial and Insurance Support	5.21%	0.720	0.29
Factor 5: Attitudes about Work	6.19%	0.803	0.66
Factor 6: Workplace, Economic and External Factors	4.91%	0.619	0.03
Factor 7: Meaning of Work	4.02%	0.729	0.96
Total Scale	66.45%	0.787	0.62

**Table 4:** Accuracy Measures for CAWSE Scores Using Varying Cut-Offs.

Cut-Off Range	Sensitivity (%)	1-Specificity (%)
99.5 vs. 100.5	89.7	18.6
101.5 vs. 102.5	89.1	23.3
104.5 vs. 106.5	87.9	27.9
108.5 vs. 110.5	85.1	27.9
112.5 vs. 115.0	83.3	27.9
117.0 vs. 118.5	81.6	27.9
120.5 vs. 123.5	79.9	37.2
125.5 vs. 126.5	78.2	41.9
130.5 vs. 132.5	75.3	46.5
133.5 vs. 135.5	69.5	55.8

## Data Availability

The data presented in this study are available upon reasonable request from the corresponding author. Due to the sensitive nature of health and vocational implications, as well as the potential risks of bias and stigmatization, access to the data are restricted.

## Supplementary Materials

The following supporting information can be downloaded at: https://www.mdpi.com/article/10.3390/curroncol32030166/s1, Supplemental Document S1: CAWSE.

## Abbreviations

CAWSE	Cancer and Work Scale
RTW	Return to Work
ITBC	Individuals Touched by Cancer
EFA	Exploratory Factor Analysis
CFA	Confirmatory Factor Analysis
AUC	Area Under the Curve
HRQoL	Health-Related Quality of Life
COSMIN	Consensus-Based Standards for the Selection of Health Measurement Instruments
HADS	Hospital Anxiety and Depression Scale
EORTC QLQ-C30	European Organisation for Research and Treatment of Cancer Quality of Life Questionnaire
