# Supplementary material for: The Cancer and Work Scale (CAWSE): Assessing Return to Work Likelihood and Employment Sustainability After Cancer
Source: Curr Oncol. 2025 Mar 14;32(3):166. doi: 10.3390/curroncol32030166 (PMC11940880; doi:10.3390/curroncol32030166)
Supplement: Supplementary file 1 [file curroncol-32-00166-s001.zip › curroncol-3485249-supplementary.pdf]

CAWSE<br>The Cancer and Work Scale

Purpose: The **Cancer and Work Scale (CAWSE)** assesses an individual's likelihood of sustaining employment following a cancer diagnosis, whether they have already returned to work or have not yet resumed employment. It evaluates factors influencing return to work, the ability to maintain employment, and adjustments to working hours for those who have temporarily reduced their workload due to cancer. To ensure relevance across different work statuses, CAWSE items should be interpreted based on the individual's current situation. Those who have not yet returned to work should consider items in relation to their anticipated experience, while those who have already returned should interpret them in the present tense, reflecting their ongoing employment.

Scoring: **CAWSE** is a 31-item scale grouped into 7 subscales: 1. Coping and Well-Being at Work (7 items) 2. Perceived Impact of Cancer on Work (4 items) 3. Support, Communication, and Accommodations at Work (5 items) 4. Financial and Insurance Support (4 items) 5. Attitudes About Work (5 items) 6. Workplace, Economic, and External Factors (3 items) 7. Meaning of Work (3 items). Each subscale is rated on a 7-point Likert scale ranging from 1 ("strongly disagree") to 7 ("strongly agree"). Reverse coding is required for specific items (items 8, 9, 10, 11, 24, 25, and 30). In reverse coding, a score of 7 is re-coded as 1, 6 as 2, 5 as 3, and 4 remains unchanged.
To calculate the total score, sum the scores for all 31 items. The possible total score ranges from 31 (minimum) to 217 (maximum).

Interpretation: A total score of 123 or below indicates a lower likelihood of sustaining employment and suggests the need for targeted professional support to sustain or improve work engagement.
The final unscored item, if answered "yes," highlights additional support needs, although it is not included in the total score calculation.

Scale Instructions: Please mark an **"X"** in the box that best represents your response to the following statements.
Note: If you have already returned to work, interpret items as referring to your current work experience. If you have not yet returned to work, read items referring to your anticipated work experience. For example:If you have not yet returned to work, interpret the item *"Working would make me feel like life goes on."*. If you have already returned to work, interpret the item as *"Working makes me feel like life goes on."*

Use this approach throughout the questionnaire to ensure your responses accurately reflect your situation. The ranking system is as follows:

1	2	3	4	5	6	7
Strongly Disagree	Disagree	Somewhat Disagree	Neutral	Somewhat Agree	Agree	Strongly Agree

**F1. Coping and Well-being at Work**	**1**	**2**	**3**	**4**	**5**	**6**	**7**
1. Working would make me feel like life goes on.							
2. Being at work would help me in my recovery.							
3. Working keeps me from getting caught up in my thoughts.							
4. Working would make me feel normal again.							
5. Working gives me a sense of purpose.							
6. Working is a distraction from my cancer.							
7. The routine of working is likely to help me get better.							
**F2. Perceived Impact of Cancer on Work**	**1**	**2**	**3**	**4**	**5**	**6**	**7**
8. Feeling tired most days is likely to affect my work.							
9. Problems with my memory are likely to affect my work.							
10. The physical demands of work are likely to make working more difficult.							
11. Difficulties with my concentration are likely to affect my work.							
**F3. Support, Communication, and Accommodations at Work**	**1**	**2**	**3**	**4**	**5**	**6**	**7**
12. My job supervisor is considerate of my cancer and how it affects my work.							
13. My colleagues are considerate of my cancer and how it affects my work.							

	1	2	3	4	5	6	7
14. My job supervisor tries to make things easier for me at work.							
15. I can communicate openly with my supervisor about my cancer and how it affects my work.							
16. My work has provided me with flexible working conditions.							
**F4. Financial and Insurance Support**	**1**	**2**	**3**	**4**	**5**	**6**	**7**
17. I need to work because I depend on my income.							
18. I have returned to work or am considering returning because I am afraid of losing my job permanently.							
19. I need to work because I depend on my pension.							
20. I need to work because I depend on my medical insurance.							
**F5: Attitudes About Work**	**1**	**2**	**3**	**4**	**5**	**6**	**7**
21. I really enjoy my work.							
22. My job is varied and interesting.							
23. I still enjoy my work just as much as I did before I was diagnosed with cancer.							
24. I no longer feel needed at work.							
25. I like work less than I did before I was diagnosed with cancer.							
**F6: Workplace, Economic, and External Factors**	**1**	**2**	**3**	**4**	**5**	**6**	**7**
26. I felt pressure from my family to return to work.							
27. I was afraid of missing out on opportunities for promotions if I did not go back to work.							
28. I felt pressure from my friends to return to work.							
**F7: Meaning of Work**	**1**	**2**	**3**	**4**	**5**	**6**	**7**
29. I would feel lonely if I was not working.							
30. Life still has meaning, even if I do not work.							
31. If I was not working, I would not know what to do with my time.							

**Final Question**	**Yes**	**No**
Would you be interested in speaking with a professional to receive guidance, whether you are currently working or not, for sustaining employment, including a return to work, and maintaining working hours?		

Thank you for completing the **Cancer and Work Scale** (CAWSE). The responses provided in each section and question can assist in designing targeted and individualized strategies to support sustained employment after cancer, including a return to work.

Reference: Maheu, C.; Singh, M.; Tock, W.L.; Robert, J.; Vodermaier, A.; Parkinson, M.; Dolgoy, N. The Cancer and Work Scale (CAWSE): Assessing Return to Work Likelihood and Employment Sustainability After Cancer. Curr. Oncol. 2025, 32, x. https://doi.org/10.3390/xxxxx

Also available on the Cancer and Work website www.cancerandwork.ca
